# Treatment of Chronic Urethritis

**Published:** 1886-08

**Authors:** 


### *TREATMENT OF CHRONIC URETHRITIS.*

Dr. O. D. Ball gives, in the Albany *Medical Annals*, a new method of treating chronic urethral discharges. His plan consists in the local application of zinc ointment to the urethral mucous membrane. The following is his formula:

℞ Zinci Oxidi, - - - - ʒiii.

Adipis, - - - - - - ʒiii.

Cerati Simp., - - - - ʒii.

M. Sig. Ointment.

This is applied by means of an olive-headed bougie. The constriction of the bougie is filled with the ointment and rapidly carried down to prostatic urethra, where it is rotated and slowly withdrawn. Dr. Ball has treated fifteen cases with this remedy—all successfully. The average time of treatment was a little over four weeks.

